# HIF-1–dependent regulation of lifespan in *Caenorhabditis elegans* by the acyl-CoA–binding protein MAA-1

**DOI:** 10.18632/aging.101267

**Published:** 2017-07-27

**Authors:** Mehrnaz Shamalnasab, Manel Dhaoui, Manjunatha Thondamal, Eva Bang Harvald, Nils J. Færgeman, Hugo Aguilaniu, Paola Fabrizio

**Affiliations:** ^1^ Institut de Génomique Fonctionnelle de Lyon, Centre National de la Recherche Scientifique, Université de Lyon 1, Ecole Normale Supérieure, Lyon, France; ^2^ Villum Center for Bioanalytical Sciences, Department of Biochemistry and Molecular Biology, University of Southern Denmark, Odense M, Denmark

**Keywords:** *C. elegans*, ACBP, HIF-1, aging, proteostasis

## Abstract

In yeast, the broadly conserved acyl-CoA–binding protein (ACBP) is a negative regulator of stress resistance and longevity. Here, we have turned to the nematode *C. elegans* as a model organism in which to determine whether ACBPs play similar roles in multicellular organisms. We systematically inactivated each of the seven *C. elegans* ACBP paralogs and found that one of them, *maa-1* (which encodes membrane-associated ACBP 1), is indeed involved in the regulation of longevity. In fact, loss of *maa-1* promotes lifespan extension and resistance to different types of stress. Through genetic and gene expression studies we have demonstrated that HIF-1, a master transcriptional regulator of adaptation to hypoxia, plays a central role in orchestrating the anti-aging response induced by MAA-1 deficiency. This response relies on the activation of molecular chaperones known to contribute to maintenance of the proteome. Our work extends to *C. elegans* the role of ACBP in aging, implicates HIF-1 in the increase of lifespan of *maa-1* –deficient worms, and sheds light on the anti-aging function of HIF-1. Given that both ACBP and HIF-1 are highly conserved, our results suggest the possible involvement of these proteins in the age-associated decline in proteostasis in mammals.

## INTRODUCTION

Genome-wide screens in simple model organisms have identified a number of longevity genes with potentially conserved roles in aging in mammals. The validity of this approach is supported by work in the last two decades showing that the principal lifespan-regulating genes and pathways are conserved in species ranging from yeast to mice [1].

Several novel lifespan determinants have previously been identified by screening for all viable yeast deletion mutants [2]. Deletion of one of the genes identified, *ACB1*, not only doubles the mean lifespan of yeast but also markedly enhances heat resistance, a phenotype often associated with extended longevity in yeast and worms [2]. *ACB1* encodes the highly conserved acyl-CoA–binding protein (ACBP), which is found in all eukaryotes and some prokaryotes tested to date [3]. ACBP binds with high affinity and specificity to long- and medium-chain acyl-CoA esters and is thought to protect them from hydrolysis during transport to acyl-CoA consuming processes such as lipid biosynthesis and remodeling, β-oxidation, and protein acylation [4]. Acb1, the only ACBP in *S. cerevisiae*, has been studied extensively. Its depletion has been shown to retard growth and reduce sphingolipid biosynthesis [5], and the observations that Acb1 deficiency perturbs plasma membrane structure, disrupts vacuole assembly, and causes vesicle accumulation suggest a key role for Acb1 in vesicular trafficking and membrane assembly [5, 6].

Although mammals express several ACBP paralogs with varying numbers of functional domains, the majority of studies have focused on the ubiquitously expressed ACBD1 [7]. In both human and bovine epithelial cells, ACBD1 is predominantly localized in the cytosol but is also present in the endoplasmic reticulum (ER), Golgi, and nucleus [8]. ACBD1 knockout mice are fertile and develop normally, but show a delay in the induction of liver lipogenesis at weaning [9]. This is consistent with studies of cultured cells pointing to a role for ACBP in the promotion of adipocyte differentiation, metabolism of triacyl-glycerides (TAGs), and activation of lipid biosynthetic genes [10-12].

The *C. elegans* genome encodes seven ACBP paralogs. Four of these, ACBP-1, -3, -4, and -6, contain only the ACBP domain, while ACBP-2, -5, and MAA-1 carry additional domains [13]. Ectopic expression of each *C. elegans* paralog can complement the slow growth of yeast *acb1* deletion mutants, suggesting that the *C. elegans* ACBPs are functional acyl-CoA–binding proteins [13, 14]. However, the paralogs have different expression patterns depending on the developmental stage and/or the tissue examined, suggesting they may each have distinct or only partially overlapping functions [13]. ACBP-2, which contains an enoyl-CoA hydratase/isomerase (ECH) domain, plays an important role in promoting β-oxidation of unsaturated fatty acids [13]. Compared with wild-type worms, ACBP-1–deficient animals contain lower TAG levels and fewer but larger lipid droplets in the intestine [13]. MAA-1 (membrane-associated ACBP 1) is a transmembrane protein detected exclusively in the intestine and hypodermal cells, where it localizes to the Golgi and to the endocytic recycling compartment (ERC) [14]. Loss of *maa-1* reduces endocytic recycling rates and alters the morphology of the ERC membrane, consistent with a role for MAA-1 in recruiting long-chain acyl-CoA esters to the endosomal and Golgi systems to promote vesicle fission or fusion [14]. Notably, long-chain acyl-CoA esters are required for Golgi vesicle fission/fusion *in vitro*, although their function has yet to be elucidated [15, 16]. A role for MAA-1 in vesicular trafficking is consistent with one of the proposed roles for yeast Acb1 [5, 6] suggesting that MAA-1 in the *C. elegans* intestine and hypodermis may have evolved to control the formation of an intramembrane acyl-CoA ester pool required for vesicle formation [14]. Taken together, studies of ACBP paralogs in different species have revealed a complex functional repertoire for ACBPs in processes ranging from lipogenesis to vesicle trafficking.

The high interspecies conservation of ACBP and the discovery that yeast Acb1 controls longevity prompted us to investigate the role(s) of ACBP in lifespan regulation in a multicellular organism. We found that of the seven *C. elegans* paralogs, *maa-1* alone played a significant role in regulating lifespan. MAA-1 deficiency prolonged lifespan through a mechanism involving the hypoxia inducible factor 1 (HIF-1), which has recently been shown to play dual roles in limiting and extending the *C. elegans* lifespan through distinct, but as yet not fully characterized, mechanisms [17-20]. In this study, we identified an ACBP- and HIF-1–dependent pathway for longevity regulation in *C. elegans* that involves small heat-shock proteins and functions under hypoxia-independent conditions.

## RESULTS

### *maa-1* inactivation promotes lifespan extension and stress resistance in *C. elegans*

**Figure 1:**
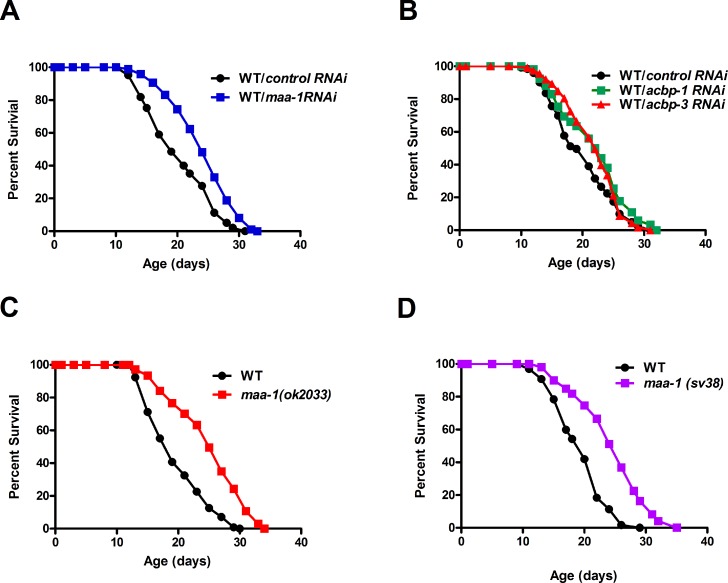
*ACBPs* regulate *C. elegans* lifespan (**A**) Downregulation of *maa-1* promotes longevity. Lifespans of wildtype worms (N2) subjected to control RNAi vs *maa-1* dsRNA, P<0.0001. (**B**) Downregulation of *acbp-1* or *acbp-3* modestly increases longevity. Lifespans of wild-type worms subjected to control RNAi vs *acbp-1* RNAi, P<0.01; and control RNAi vs *acbp-3* RNAi, P=0.056. (C-D) Loss-of-function mutations *maa-1(ok2033)* (**C**) and *maa-1(sv38)* (**D**) prolong lifespan (both P<0.0001). P values were calculated using the log-rank (Mantel-Cox) method. Replicate experiments and details of statistical analyses are shown in Table S1 and S2.

An earlier study failed to show any extension of lifespan in a few ACBP null mutants of *C. elegans* [13]. Since complete loss of ACBP causes growth defects in yeast, we reasoned that a reduction, rather than a total loss, of gene function might reveal a contribution of ACBP to lifespan in *C. elegans*. To address this, we used dsRNA-mediated RNAi to individually down-regulate each of the seven ACBP genes. Indeed, the lifespan of wild-type worms was increased markedly by knockdown of *maa-1* (Figure 1A; Table S1 and S2) and more modestly by knockdown of either *acpb-1* or *acbp-3* (Figure 1B; Table S1). We confirmed the effect of *maa-1* deficiency on longevity using two *maa-1* deletion mutants (*ok2033* and *sv38*) (Figure 1C,D and S1). Only the *sv38* allele had previously been established to be null [14]; however, both mutants showed similar survival curves, suggesting that *maa-1*(*ok2033)* may also be a null allele (Figure 1C,D; Table S1 and S2). Given that manipulation of *maa-1* expression has the most marked effects on *C. elegans* lifespan, we focused our further analyses on this ACBP paralog.

**Figure 2:**
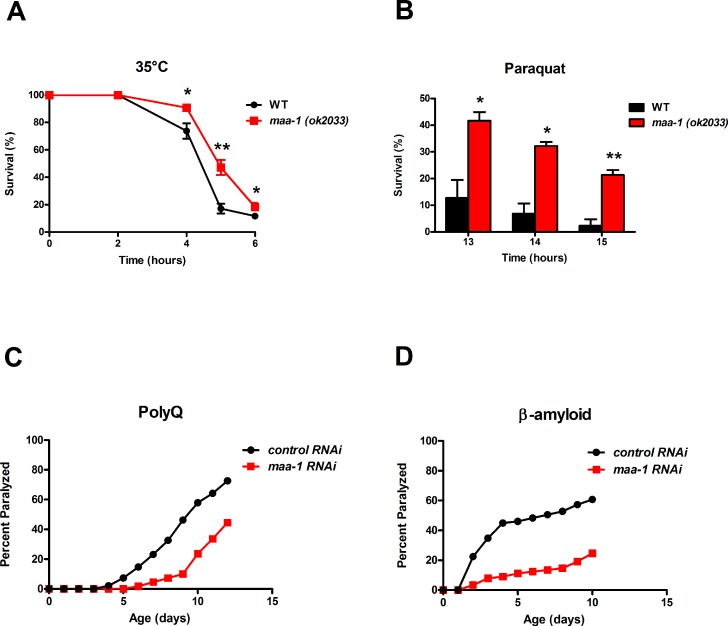
Loss of *maa‐1* promotes heat, oxidative, and proteotoxic stress resistance **A**‐**B**) *maa‐1(ok2033)* mutants showed enhanced resistance to incubation at 35°C (**A**) and exposure to 150 mM paraquat (**B**). Error bars represent SEM from three independent experiments (*P<0.05, **P<0.01 compared to wildtype worms by Student's t‐test). (**C**‐**D**) *maa‐1* RNAi increases resistance to paralysis induced by aggregation of a 35‐residue polyglutamine repeat (**C**; P<0.0001) or human β‐amyloid (**D**; P<0.0001). P values were calculated using the log‐rank (Mantel‐Cox) method. Replicate experiment is shown in Figure S2.

Several lifespan-extending mutations, including deletion of *ACB1* in yeast, concomitantly increase resistance to various forms of stress [1]. Consistent with this, we found that *maa-1 (ok2033)* mutants were more resistant than wild-type animals to both thermal stress induced by incubation at 35°C and oxidative stress induced by the superoxide generator paraquat (Figure 2A,B). Stress induced by protein misfolding and aggregation is thought to contribute to the aging process in many species, and elevated resistance to such proteotoxicity is another feature common to many long-lived *C. elegans* mutants [21]. Several transgenic models of proteotoxicity have been developed in *C. elegans*, two of which are expression of polyglutamine repeats fused to YFP (Q_35_YFP) or human β-amyloid peptide (AΔ_1-42_) in the body wall muscle. In these animals, the effect of proteotoxic stress is conveniently measured as age-dependent paralysis [22, 23]. We found that in both of these models, loss of motility was significantly delayed by *maa-1* RNAi (Figures 2C,D and S2A,B), suggesting that loss of MAA-1 activity counteracts the age-associated disruption of proteostasis. Collectively, these data indicate a novel role for MAA-1 in stress resistance and longevity in *C. elegans*.

### MAA-1 functions in the intestine to control longevity

**Figure 3:**
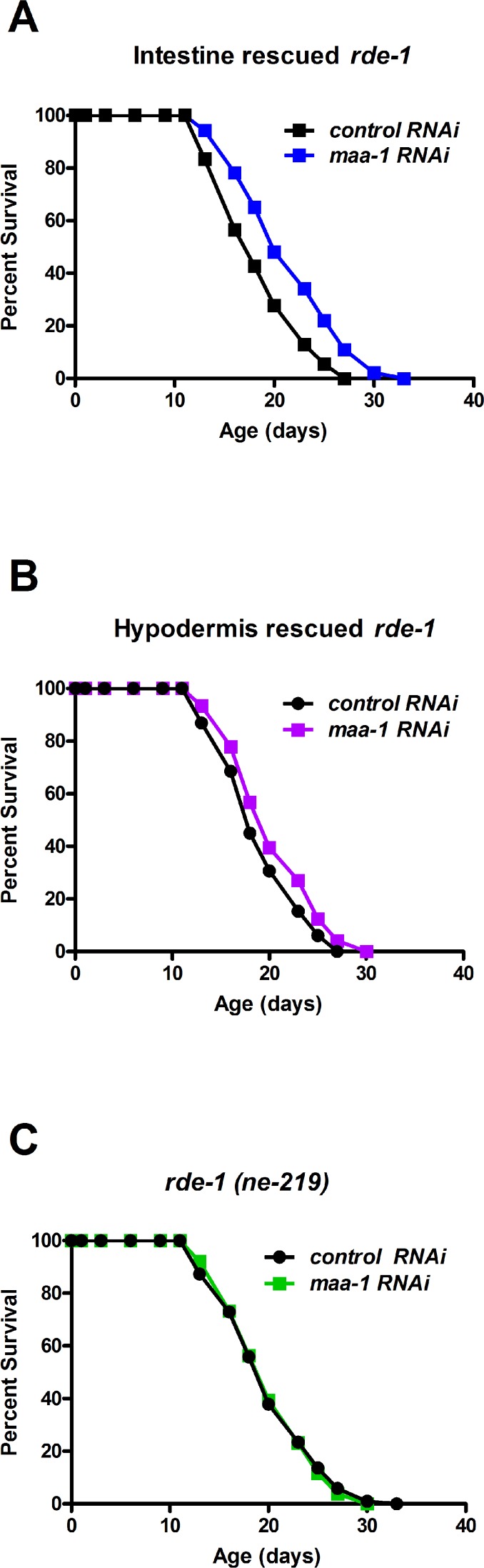
MAA-1 functions predominantly in the intestine to regulate longevity Intestinal-specific RNAi is sufficient to extend longevity. Lifespans of *rde-1(ne219)* mutants in which *rde-1* expression is restored in the intestine (**A**) or the hypodermis (**B**); animals were subjected to control or *maa-1* RNAi (P<0.0001 and P<0.05 for **A** and **B**, respectively). (**C**) Lifespans of the control strain *rde-1(ne219)* subjected to control or *maa-1* RNAi (P=0.8513). P values were calculated using the log-rank (Mantel-Cox) method. Replicate experiments are shown and additional statistical analysis are shown in Table S1 and S2.

Previous studies of *C. elegans* lines expressing a MAA-1::GFP fusion protein have shown that MAA-1 is highly expressed in intestinal and hypodermal cells, but is essentially undetectable elsewhere [14]. To identify the tissue(s) in which MAA-1 functions to regulate lifespan, we performed tissue-specific RNAi. RDE-1 is an essential component of the RNAi machinery and inactivation abolishes the RNAi response in all tissues. Re-expressing *rde-1*(+) under the control of a tissue-specific promoter therefore allows the effects of RNAi to be examined specifically in that tissue. To reduce *maa-1* expression exclusively in the intestine or hypodermis, we used *rde-1(ne129)* mutants in which *rde-1*(+) had been restored using the *elt-2* (intestine) and *lin-26* (hypodermis) promoters (R. Roy, un-published results and [24]). *rde-1(ne129)* mutants, in which RNAi is inactivated ubiquitously, were used as control animals. Down-regulation of *maa-1* in the intestine resulted in a highly significant longevity extension (Figure 3A; Table S1 and S2). Conversely, the hypodermal down-regulation of *maa-1* had only a minor effect on longevity (Figure 3B) and, as expected, *maa-1* RNAi had no effect on the lifespan of the unrescued *rde-1(ne129)* mutants (Figure 3C; Table S1).

To further substantiate the modest response to hypodermal down-regulation of *maa-1*, we used an alternative transgenic animal in which the *wrt-2* promoter was used to rescue the *rde-1*(+) function exclusively in the hypodermis [25]. The results were consistent with those obtained with the *lin-26p::rde-1* transgenic line (Figure S3, Table S1 and S2). Together, these results suggest that MAA-1 functions to modulate aging predominantly in the intestine and to a much lesser extent in the hypodermis.

### HIF-1 mediates lifespan extension and proteotoxic stress resistance induced by *maa-1* inactivation

As shown above (Figure 2C,D), *maa-1* RNAi protects worms against age-dependent proteotoxicity. Since a prominent role in proteome maintenance and lifespan regulation has recently emerged for HIF-1 [26], a highly conserved transcriptional regulator of the metazoan response to hypoxia [27], we investigated a possible role for *hif-1* in extending the lifespan of *maa-1–*deficient animals.

**Figure 4:**
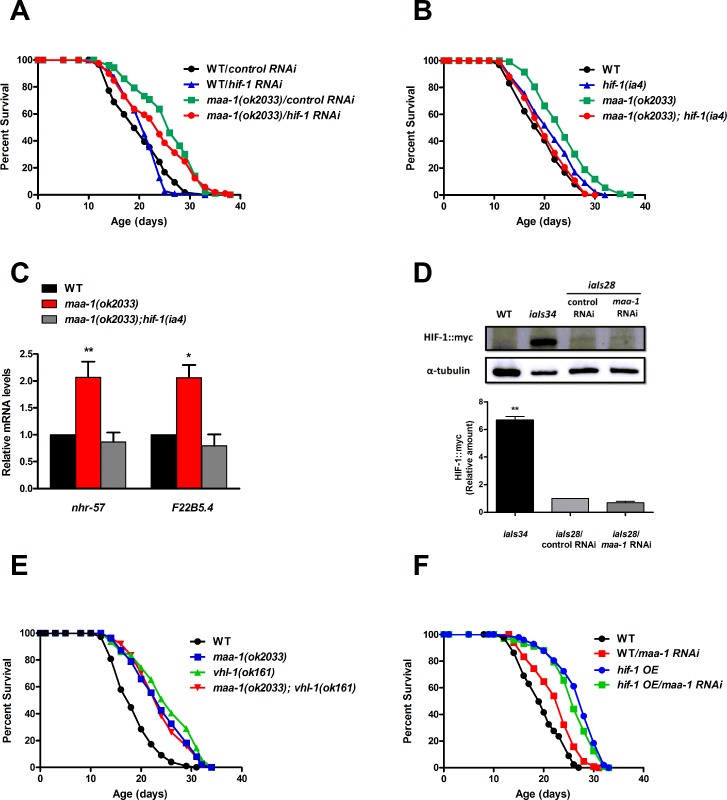
HIF‐1 mediates lifespan extension of *maa‐1*–deficient animals (**A**) Downregulation of *hif‐1* reduces the longevity of *maa‐ 1(ok2033)* mutants. Lifespans of wild‐type and *maa‐1(ok2033)* mutants subjected to *hif‐1* or control RNAi (P=0.1091 for control RNAi vs *hif‐1* RNAi of *maa‐1(ok2033)* mutants). (**B**) Deletion of *hif‐1* reverses the lifespan extension conferred by the *maa‐1* mutation. (P=0.2661 for wild‐type vs *maa‐1(ok2033); hif‐1(ia4)* mutants; P<0.0001 for *maa‐1(ok2033)* vs *maa‐1(ok2033); hif‐1(ia4)* mutants). (**C**) qPCR analysis of the HIF‐1 targets *nhr‐57* and *F22B5.4* in *maa‐1(ok2033)* and *maa‐1(ok2033); hif‐1(ia4)* mutants. Results are relative to levels in wildtype animals. Error bars represent SEM (t‐test: *P<0.05, **P<0.001 for *maa‐1(ok2033)* vs wildtype animals). (**D**) HIF‐1 stability is not affected by downregulation of *maa‐1*. Western blot of protein extracts from wildtype, transgenic *iaIs34* animals carrying HIF‐1 (P621G)::myc, and transgenic *iaIs28* animals carrying HIF‐1::myc, subjected to control or *maa‐1* RNAi. Blots were probed with anti‐myc and anti‐α‐tubulin antibodies (upper panel). Quantification of band intensity is shown in lower panel (N=3, **P<0.001). (**E**) A *maa‐1* loss‐of‐function mutation does not further increase the lifespan of long‐lived *vhl‐1(ok161)* mutants (P=0.0692 for *vhl‐1(ok161)* vs *maa‐1(ok2033); vhl‐1(ok161)*, P=0.8449 for *maa‐1(ok2033)* vs *maa‐1(ok2033); vhl‐1(ok161)*). (**F**) Downregulation of *maa‐1* does not affect the lifespan of long‐lived transgenic animals overexpressing HIF‐1(P621G)::myc (*hif‐1 OE*). (P=0.0678 for *hif‐1 OE* on control vs *maa‐1* RNAi). P values were calculated using the log‐rank (Mantel‐Cox) method. Replicate experiments are shown in Table S1. Additional statistical analysis is shown in Table S2.

Whereas *hif-1* RNAi had no effect on the lifespan of wildtype animals, it partially suppressed the lifespan extension conferred by the *maa-1(ok2033)* allele (Figure 4A; Table S1). We then generated double mutants combining the *maa-1(ok2033)* and loss-of-function *hif-1(ia4)* alleles, and observed complete reversion of the longevity phenotype of the *maa-1 (ok2033) single* mutants (Figure 4B; Table S1 and S2). Together, these results suggest a role for *hif-1* in prolonging the lifespan of *maa-1*–deficient animals.

Interestingly, the mean lifespan of the *hif-1(ia4)* single mutant was significantly extended in each of three independent experiments (Figure 4B and Table S1 and S2) consistently with previous work showing a negative effect of *hif-1* on longevity, which is observed most frequently at 25°C but also at 20°C [19, 20, 28]. In our studies (all performed at 20°C) the pro-longevity effect of the *hif-1(ia4)* allele is lost in the *maa-1(ok2033)*; *hif-1(ia4)* double mutant (Figure 4B and Table S1 and S2) suggesting that lack of *maa-1* inhibits the anti-aging mechanisms triggered by inactivation of *hif-1*.

To determine whether HIF-1 transcriptional activity was necessary for its function in *maa-1* mutant animals, we analyzed the expression of three known HIF-1-dependent target genes induced by hypoxia; *nhr-57*, *F22B5.4*, and *fmo-2*. The latter, *fmo-2*, codes for the xenobiotic detoxification enzyme flavin containing monoxygenase-2, which plays a key role in promoting longevity in response to HIF-1 stabilization [29]. Notably, two of the genes tested, *nhr-57* and *F22B5.4*, were significantly up-regulated in *maa-1(ok2033)* mutants compared to wild-type animals, and the induction was *hif-1* dependent (Figure 4C and data not shown). These data are in agreement with an inhibitory role for MAA-1 in the regulation of HIF-1–driven transcription. Because HIF-1 transcriptional activity is tightly regulated by changes in its stability, we asked whether *maa-1* RNAi might increase HIF-1 protein levels. To test this, we analyzed a transgenic line carrying integrated copies of myc-tagged *hif-1 (iaIs28)* [19]. However, we observed no differences between HIF-1∷myc proteins levels in animals subjected to control and *maa-1* RNAi, ruling out a major role for MAA-1 in controlling HIF-1 protein stability (Figure 4D).

Previous work has described a role for *hif-1* in the long lifespan of animals carrying a mutation in *vhl-1*, the *C. elegans* homolog of the mammalian von Hippel-Lindau *VHL* tumor suppressor gene. Mutants carrying the loss-of-function *vhl-1(ok161)* allele live longer than wild-type worms (Figure 4E and [17, 18]), and the effect is fully dependent on HIF-1 activity [17]. To further investigate the role of HIF-1 in the longevity of *maa-1* mutants, we analyzed the genetic interaction between *maa-1* and *vhl-1*. However, we found that each single mutant and the *maa-1(ok2033)*; *vhl-1(ok161)* double mutants showed similarly extended lifespans, suggesting that both *maa-1* and *vhl-1* function through *hif-1* to modulate longevity (Figure 4E; Table S1 and S2). Finally, we performed *maa-1* RNAi on a long-lived transgenic line overexpressing HIF-1 (P621G), a stabilized version of HIF-1 [19]. Consistent with the results of experiments with the genetic mutants, this manipulation had no significant effect on the lifespan of HIF-1–overexpressing animals, confirming that HIF-1 is a key mediator of the effect of *maa-1* on longevity (Figure 4F; Table S1 and S2).

Overall, these results suggest a requirement for HIF-1 transcriptional activity in extending the lifespan of the *maa-1 (ok2033) via* mechanisms independent of both HIF-1 stability and *fmo*-2 expression.

To test whether HIF-1 was responsible for the enhanced resistance to proteotoxicty in response to *maa-1* deficiency we generated β-amyloid peptide (AΔ_1-42_) transgenic animals carrying the *hif-1 (ia4)* null allele. In contrast with the control line, transgenic worms lacking *hif-1* did not show any improvement in age-dependent paralysis when subjected to *maa-1* RNAi (Figure S4A,B) pointing to an important role for HIF-1 in mediating the effect of loss of *maa-1* on proteotoxic stress.

### DAF-16 is required for lifespan extension induced by maa-1 inactivation

**Figure 5:**
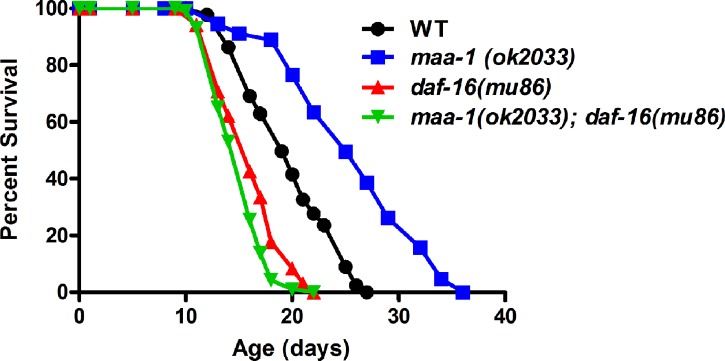
DAF-16 is required for lifespan extension in *maa-1*–deficient animals Lifespan of *maa-1(ok2033); daf-16(mu86)* double mutants is significantly shorter than that of *maa-1(ok2033)* single mutants (P<0.0001). P values were calculated using the log-rank (Mantel-Cox) method. Replicate experiments and additional statistical analysis are shown in Table S1 and S2.

To identify additional mediators of longevity extension downstream of *maa-1*, we examined the contribution of the forkhead transcription factor DAF-16, a master regulator of longevity in nematodes. DAF-16 nuclear translocation is induced in mutants with reduced insulin/IGF-1-like signaling (IIS) and mediates their longevity response [30-32]. Similarly, DAF-16 transcriptional activity contributes to enhanced longevity induced by germline disruption, TOR inactivation, or temperature reduction [32-34]. To investigate whether *daf-16* might be involved in the longevity phenotype of *maa-1* mutants, we generated *maa-1 (ok2033); daf-16 (mu86)* double mutants. The lifespan of *daf-16(mu86)* mutants was shorter than that of wildtype animals, as expected; however, we found that *daf-16* deletion completely eliminated the lifespan extension conferred by loss of *maa-1* (Figure 5; Table S1 and S2), pointing to a role for *daf-16* in mediating the effect of *maa-1* on longevity. To test this further, we asked whether DAF-16 nuclear translocation was affected by the loss of *maa-1*. However, nuclear localization of DAF-16::GFP in *maa-1(ok2033)* mutants carrying a *daf-16::GFP* transgene was comparable to that of wildtype *daf-16::GFP* animals under standard conditions (Figure S5A). As expected, DAF-16::GFP relocation was increased in both wildtype and *maa-1(ok2033)* worms subjected to heat-shock at 37°C, demonstrating that DAF-16 is functional in the *maa-1(ok2033)* mutants (Figure S5A).

We next asked whether loss of *maa-1* might increase DAF-16 transcriptional activity independently of nuclear translocation [33, 35-37]. For this, we examined the expression of *sod-3*, a well-established transcript-tional target of DAF-16 [38]. However, *sod-3* mRNA level was not increased in *maa-1(ok2033)* mutants compared to wild-type worms (Figure S5B). Together, these results suggest that although DAF-16 is required for longevity extension induced by *maa-1* deficiency, neither its nuclear translocation nor its transcriptional activity is affected by *maa-1* inactivation.

### Small heat-shock proteins modulate longevity in maa-1 deficient worms

Although the effects of HIF-1 activity on *C. elegans* longevity have been reported by several laboratories [26], the HIF-1–dependent transcriptional program responsible for lifespan modulation is not fully understood.

Recent work has shown that the survival of AMPK null mutant dauer larvae is improved by increases in total triglyceride levels and qualitative alterations in fatty acid content triggered by the stabilization of HIF-1 [39]. However, we found no differences in either the total lipid content or the fatty acid content of wild-type and *maa-1(ok2033)* mutants (Figures S6 and S7), ruling out a role for fatty acid biosynthesis in the extended lifespan of *maa-1*–deficient worms.

HIF-1 has also been reported to activate the *C. elegans* ER-associated unfolded protein response (UPR^ER^) [29, 40], a compartment-specific stress response implicated in lifespan regulation [41]. We tested the contribution of UPR^ER^ activation to the longevity of *maa-1* mutants by examining their resistance to the ER stress inducer tunicamycin. For this, worms at the L4 larval stage were grown to adulthood on tunicamycin-containing plates, and the development and survival of their progeny was monitored for 72 h. We observed no difference between *maa-1* mutants and wildtype animals in the number of eggs reaching adulthood (Figure S8). Although not conclusive, our results do not support a role for the UPR^ER^ in mediating longevity extension in *maa-1*–deficient worms.

**Figure 6:**
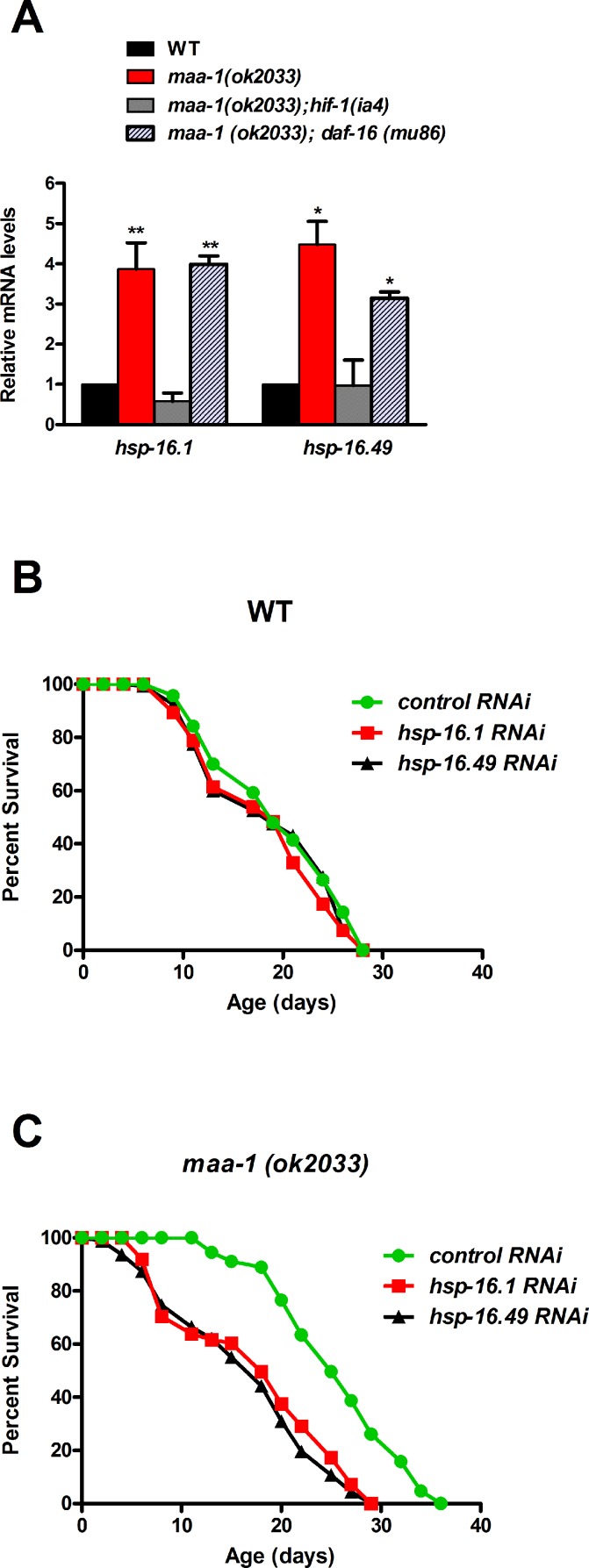
Molecular chaperones promote longevity downstream of HIF‐1 in *maa‐1–*deficient mutants (**A**) The expression of two s*hsp* genes is induced by *maa‐1* deficiency. qPCR analysis of *hsp‐16.1* and *hsp‐16.49* in wildtype, *maa‐1(ok2033), maa‐1(ok2033); hif‐1(ia4)*, and *maa‐1(ok2033); daf‐16 (mu86)* animals. mRNA levels are expressed relative to those in wildtype animals (t‐test: *P<0.05 and **P<0.001 for *maa‐1*(ok2033) and *maa‐1(ok2033); daf‐16 (mu86)* vs wildtype) respectively. (**B**) Downregulation of *shsp* expression has little effect on the lifespan of wildtype animals (P=0.1024 and P=0.2988 for control vs *hsp‐16.1* and *hsp‐16.49,* respectively*).* (**C**) Downregulation of *shsp* expression markedly shortens the lifespan of *maa‐1(ok2033)* mutants (P<0.0001 for control vs each RNAi). P values for lifespan analyses were calculated using the log‐rank (Mantel‐Cox) method. Replicate experiments and additional statistical analysis are shown in Table S1 and S2.

To identify further mediators responsible for extending longevity in response to *maa-1* inactivation, we turned to the molecular chaperone family of small heat-shock proteins (sHSPs). sHSPs delay the aggregation of poly-glutamine repeat-containing proteins and contribute to the longevity extension of IIS-deficient worms [42]. We thus asked whether *shsp* genes might be transcriptional targets of HIF-1 and play roles in extending the lifespan of *maa-1*–deficient worms. We analyzed four *shsp* genes previously shown to affect lifespan and proteostasis [42]; namely, *hsp-12.6*, *hsp-16.1*, *hsp-16.49*, and *sip-1*. Expression of *hsp-16.1* and *hsp-16.49*, but not of the other two*,* was markedly increased in *maa-1(ok2033)* mutants compared with wild-type worms, and the increases were completely reversed by concomitant deletion of *hif-1* (Figure 6A and data not shown) but not of *daf-16* (Figure 6A). These findings suggested that HIF-1–dependent transcription of *shsp* genes may play a direct role in the longevity of *maa-1* mutants.

To investigate this possibility, we assessed the lifespans of wild-type and *maa-1(ok2033)* mutants subjected to *shsp* RNAi. Down-regulation of the individual *hsp-16.1* and *hsp-16.49* genes only slightly shortened the lifespan of wild-type worms (Figure 6B, Table S1 and S2), but markedly reduced that of the *maa-1(ok2033)* mutants, strongly suggesting that, while contributing marginally to wild type lifespan, sHSPs may play significant roles downstream of HIF-1 in the control of longevity (Figure 6C, Table S1 and S2).

To further explore the role of proteostasis in extending the lifespan of *maa-1* mutants, we examined autophagy, the cellular process for recycling of cytosolic macromolecules and organelles implicated in lifespan extension in a number of species (reviewed in [43]). In *C. elegans*, autophagy is commonly monitored micro-scopically by tracking the formation of GFP-positive autophagosomes in transgenic lines expressing GFP-tagged LGG-1, the *C. elegans* homolog of the mammalian autophagy-related protein LC3. When autophagy is induced, LGG-1 localizes to the auto-phagosomal membranes, giving rise to a typical punctate staining pattern that is easily detectable in the hypodermal seam cells. We found that LGG-1::GFP transgenic animals subjected to *maa-1* RNAi exhibited a modest but significant reduction in GFP-positive autophagosomes (Figure S9), comparable to the numbers in animals subjected to RNAi of *lgg-3*, an essential autophagy gene. *let-363* (*CeTOR*) RNAi was used as positive control, because down-regulation of the TOR pathway is known to induce autophagy (Figure S9). We conclude that autophagy does not play a role in extending the lifespan of *maa-1* mutants. Furthermore, given the anti-aging role of autophagy, a reduction of autophagic activity in *maa-1* mutants might limit the beneficial effects associated with HIF-1 activation and responsible for increased longevity.

Collectively, while excluding the activation of autophagy, our results point to the HIF-1–dependent activation of small heat-shock proteins as essential to extend the lifespan of *maa-1*–deficient animals.

## DISCUSSION

In this study, we report that the *C. elegans* ACBP MAA-1 shares a conserved role in longevity regulation and stress resistance with the yeast ortholog *Acb1*. While the mechanisms by which ACBP influences yeast lifespan are not yet known, we identified several genes involved in the novel MAA-1 pathway in *C. elegans*. We found that loss of MAA-1 prolongs lifespan and promotes resistance to heat, oxidative, and proteotoxic stress; identified HIF-1 as the principal transcriptional regulator of longevity; implicated an interaction between *maa-1* and *daf-16* in lifespan regulation; and showed that HIF-1–dependent activation of *shsp* gene expression plays a key role in lifespan extension.

Recent work has demonstrated that HIF-1 stabilization in neurons is sufficient to extend worm lifespan *via* the cell non-autonomous activation of the gene coding the detoxification enzyme FMO-2 in intestinal cells, while stabilization of HIF-1 exclusively in the gut does not affect longevity [29]. The results reported here indicate that down-regulation of *maa-1* in the intestine has the major effect on lifespan (Figure 3B). This is in agreement with previous studies demonstrating a central role for intestinal cells in the regulation of longevity [44-46]. Although we can speculate about an intestinal signal leading to the neuronal activation of HIF-1 in *maa-1–*deficient worms, our results do not show HIF-1 stabilization or induction of *fmo-2* mRNA and point instead to *shsps* as key mediators of the effect of *maa-1* deficiency on lifespan. We tend therefore to favor a simpler model in which loss of intestinal *maa-1* triggers the activation of HIF-1 directly in the gut through mechanisms independent of protein stability. This activation is likely to induce both cell-autonomous and nonautonomous anti-aging mechanisms including a stress response that counteracts the toxicity of protein aggregation. Although our model may seem inconsistent with the results mentioned above [29], this apparent contradiction can be easily resolved by hypothesizing that the transcriptional response triggered by HIF-1 through protein stabilization differs in part from that induced *via* yet to be determined alternative mechanisms activated by intestinal loss of *maa-1* (see next paragraph).

How are MAA-1 and HIF-1 linked mechanistically? Of the seven ACBP genes in *C. elegans*, inactivation of *maa-1* alone markedly increased lifespan, suggesting a unique role for MAA-1 in the control of aging. Loss of MAA-1 has been proposed to alter the pool of acyl-CoA esters present on the Golgi and endosomal membranes, which are necessary for proper vesicle fusion/fission [14]. One possible direct link between MAA-1 and HIF-1 is that a specific bioactive lipid(s) produced from acyl-CoA esters on vesicular membranes might modulate HIF-1 nuclear localization and/or transcriptional activity. RHY-1 is another negative regulator of HIF-1 that is highly expressed in intestinal cells. RHY-1 is a multipass membrane protein contain-ing an acyltransferase-3 domain, with proposed roles in lipid synthesis, metabolism, and transport [47]. It is tempting to speculate that both MAA-1 and RHY-1 may function in the production of lipid species that regulate HIF-1 activation. Although computational analyses suggest that RHY-1 is present in the ER and plasma membrane [47], it will be of interest to determine whether MAA-1 and RHY-1 colocalize. The fact that neither RHY-1 nor MAA-1 substantially modify HIF-1 stability ([47] and Figure 4D) suggests that they may share a common mechanism for regulating HIF-1 transcriptional activity. Alternatively, MAA-1 and RHY-1 might independently control the metabolism of bioactive lipids that directly or indirectly affect HIF-1–dependent transcription.

The positive effect of *maa-1* inactivation on resistance to proteotoxicity prompted us to investigate the contributions of the proteostasis network to the longevity of *maa-1–*deficient mutants. We focused on the sHSP family of chaperones because of the well-established links between sHSP activation, reduced protein aggregation, and extended lifespan [42]. In *C. elegans*, the expression of various *shsp* genes is known to be regulated by the stress response transcription factors DAF-16 and HSF-1 [42]. Our data extend these observations by demonstrating HIF-1–dependent upregulation of *hsp-16.1* and *hsp-16.49*, in *maa-1*–deficient animals (Figure 6A), consistent with the suggestion that HIF-1, DAF-16, and HSF-1 have common gene targets [48]. RNAi-mediated knockdown of each of the two *shsp* genes abolished the longevity extension of *maa-1* mutants, pointing to the importance of these chaperones as key mediators of longevity downstream of HIF-1. Of note, this is a novel mechanistic explanation for the anti-aging role of HIF-1 in *C. elegans*. Interestingly, several stress response genes, including *HSP104, HSP26*, and *HSP12*, have been reported to be induced by Acb1 depletion in yeast [49]. Hsp104 and Hsp26 function together to counteract protein aggregation, and both Hsp104 and Hsp12 have reported roles in lifespan regulation [50-52]. Moreover, *HSP104, HSP26*, and *HSP12* are targets of transcription factors long known to control yeast lifespan and/or stress resistance; namely, Msn2, Msn4, Gis1, and Hsf1 [1]. It seems reasonable to assume that one or more of these transcription factors replace HIF-1 (not present in yeast) in a conserved ACBP–dependent stress response, which may function to extend longevity – at least in part – by promoting proteostasis.

Our epistasis analyses additionally show that *daf-16* is required to extend the lifespan of *maa-1*–deficient animals (Figure 5, Table S1 and S2), although paradoxically, *daf-16* nuclear localization and transcriptional activity (as indicated by expression of its direct transcriptional target *sod-3*) are unaffected by *maa-1* loss (Figure S5A,B). Notably, the expression of *hsps* triggered by *maa-1* deficiency is not reversed by loss of DAF-16 ruling out a direct contribution of DAF-16 to the activation of stress response observed. Together, our results suggest that DAF-16 basal activity is necessary but not sufficient for *maa-1(ok2033)* animals to reach their full longevity potential. Alternatively, loss of *maa-1* may induce the DAF-16–dependent transcription of a set of genes yet to be identified.

In *C. elegans* the role played by HIF-1 in lifespan regulation is complex. Several articles have demonstrated a positive effect of HIF-1 on longevity [17-19, 28]. However, worms carrying *hif-1* loss of function alleles live longer than wild type [19, 20, 28]. These paradoxical findings have not been fully understood [26]. While the results presented here and those of others [29] shed light on the anti-aging role of *hif-1*, the anti-aging mechanisms triggered by loss of *hif-1* are still elusive. Recent work has shown that lack of *hif-1* extends longevity significantly only at 25°C whereas a modest effect can be observed at 20°C only if the frequent deaths due to vulval rupture are censored [28]. Intriguingly, in the lifespan studies reported here *hif-1(ia4)* mutants showed a consistent and significant 10% mean lifespan extension at 20C° (Figure 4B; Table S1 and S2). This effect seems to be independent of the exclusion of animals showing the “exploded through vulva” phenotype, which was negligible in our experiments (data not shown). Although the origin of these discrepancies is unclear, we speculate it may be due the different experimental conditions such as the bacterial strain used or whether it is dead or alive. It has been previously shown that both *E. coli* HT115 and OP50 differentially modulate the metabolism, behavior, development and aging of worms [53] up to an extent that certain genes may have opposite effects on the lifespan of worms fed on OP50 and HT115 [54-56]. Similarly, the use of UV-killed bacteria may differentially affect the lifespan of certain mutants [57]. Most of the previously published *hif-1 (ia4)* worm lifespan experiments have been performed on UV-killed OP50 [28]. However, in our case all lifespan experiments have been done on live HT115. Thus, it is not surprising that we obtain slightly different results than previously reported.

Of note, *maa-1; hif-1* double mutants live shorter than individual *hif-1* mutants (Table S1 and S2). This suggests that loss of *maa-1* suppresses the anti-aging response triggered by inactivation of *hif-1*. A possible explanation for these results is that, in analogy with what observed for autophagy, *maa-1* down-regulation has a negative effect on yet to be established anti-aging systems induced by *hif-1* deficiency.

In mammals, HIF-1 and HIF-2 are the master regulators of the hypoxia response and have essential roles in embryonic development as well as in the physiopathology of cardiovascular diseases and cancer [27]. Increasing evidence indicates that although HIF-1 activity is protective against ischemia, HIF-1 and/or HIF-2 activation promote metabolic reprogramming, vascularization, and metastasis in the majority of human cancers [27]. HIF stability and transcriptional activity are modulated by several post-translational modifications, and numerous signaling molecules are known to control HIF-dependent transactivation in an oxygen-independent manner [58]. Given this complex scenario, it will be worth exploring the existence of a conserved mammalian stress response pathway involving ACBP and/or acyl-CoA esters, HIF-1, and molecular chaperones such as sHSPs. Mammalian ACBD1 may be a good candidate in this regard, because it is likely to share a role with MAA-1 in vesicle trafficking [8], a function that may be relevant to the regulation of HIF-1. Similarly, it will be important to test tissues/cells isolated from ACBD1 knockout mice for changes in stress resistance, HIF-1 activity, and chaperone gene expression. Notably, studies in mice have shown an age-dependent decline in the inducible HIF-driven transcription in ischemic muscles [27]. Moreover, the age-associated decline in HIF-1 activity observed in rat liver, heart, and skeletal muscle can be reversed by dietary restriction, a well-established life-extending intervention [59]. While it seems clear from studies of cancer that constitutive stabilization of HIF-1 can be detrimental to human health, the preservation of HIF-1 inducibility over time is likely to be beneficial, both to prevent the age-associated decline in tissue function and to protect against ischemia. Thus, a comprehensive understanding of the mechanisms responsible for HIF-1 function may contribute to the development of pharmacotherapeutic strategies aimed at improving human health by modulating HIF function.

## MATERIALS AND METHODS

### Nematode maintenance and strains

Strains were cultured under standard laboratory conditions [60]. The wildtype N2 (Bristol) was used as the reference strain. Strains used are listed below (name, genotype, and origin).

RB1644, *maa-1(ok2033) III*, CGC
VB1051, *maa-1(sv38) III*, Tuck Lab
AM140, *rmIs132 [unc54p::Q-35::YFP]*, CGC
CL2006 (Aβ1-42), *dvIs2 [pCL12 (unc-54p::Aβ1-42) + pRF4]*, CGC
HGA8024, *hif-1 (ia4) V*, *dvIs2 [pCL12 (unc-54p::Aβ1-42) + pRF4]*, this work
WM27, *rde-1(ne219) V*, CGC
MR0931, *rde-1(ne129) V, rrIs01 [elt-2::GFP]; Ex236 [end-3p::rde-1; elt-2p::rde-1; inx-6::GFP]*, Roy Lab
NR222, *rde-1(ne129) V, kzIs9 [pKK1260 (lin-26p::nls::GFP); (lin-26p::rde-1) + pRF6]*, CGC
JM43, *rde-1(ne219)V, Is[wrt-2p::rde-1],* Melo Lab
AD105, *daf-16(mu86) I*, CGC
TJ356, *zIs356 IV [daf-16::GFP+pRF6],* CGC
HGA8020, *maa-1(ok2033) III; daf-16(mu86) I,* this work
HGA8021, *maa-1(ok203 3) III; zIs356 IV [daf-16::GFP + pRF6],* this work
ZG31, *hif-1(ia4) V*, CGC
HGA8022, *maa-1(ok2033) III; hif-1(ia4) V*, this work
CB5602, *vhl-1(ok16) X,* CGC
HGA8023, *vhl-1(ok16) X; maa-1(ok2033) III*, this work
DA2123, *adIs2122* [*lgg-1::GFP+pRF6]*, CGC
ZG583, *iaIs34 [hif-1p::hif-1a (P621G)::myc + unc-119(+)]*, Powell-Coffman Lab
ZG580, *iaIs28 [hif-1p::hif-1a::myc + unc-119(+)],* Powell-Coffman Lab

### Lifespan analysis

Lifespan assays were conducted at 20°C according to standard protocols [61]. Worms were synchronized by bleaching and transferred to plates at the L1 stage. Worms were maintained on solid Nematode Growth Medium (NGM) containing 25 μg/ml carbenicillin and 15 μM 5-fluorouracil and seeded with *Escherichia coli* strain HT115. Animals that failed to display heat-provoked movement were scored as dead. Animals that crawled off the plates were not included in the analysis. P values were calculated using the log-rank (Mantel-Cox) method. Statistics and significance calculations for individual lifespan studies were determined using the Oasis online software [62]. Statistical analysis of experiments shown in the main text and replicate experiments are provided in Table S1. To take into account the variance between replicate experiments we performed pairwise comparisons for both mean and maximum lifespan by two tailed Student's t-test. Results are shown in Table S2.

### RNA-mediated interference

RNAi experiments were carried out by feeding worms with bacteria expressing dsRNA against the gene of interest (or control bacteria carrying the empty L4440 vector). Bacteria transformed with the appropriate vectors were grown at 37°C overnight and then seeded onto NGM plates containing carbenicillin (25 μg/ml). Expression of dsRNA was induced by the addition of 1 mM isopropyl β-D-1-thiogalactopyranoside (IPTG; Sigma) before L1 worms were added to the bacterial lawns. RNAi clones were obtained from the Ahringer collection (Source BioScience) and were verified by sequencing prior to use.

### Quantification of proteotoxicity-induced paralysis

The paralysis of worms expressing Q_35_YFP or human Aβ_1-42_ was assessed visually, as previously described [63]. Briefly, worms were tapped on the head with a platinum wire and were scored as paralyzed if the head moved but the worm failed to make forward progress on the agar surface. The assay was terminated within day 12 to avoid mistakenly scoring old worms as paralyzed [63]. P values were calculated using the log-rank (Mantel-Cox) method.

### Resistance to oxidative stress

To measure oxidative stress resistance, fifty day-1 adult worms were transferred to M9 buffer containing 150 mM paraquat (1,1-dimethyl-4,4-bipyridinium dichlo-ride; Sigma-Aldrich) and then incubated at 20°C. Live/dead worms were scored hourly. Every time point shown consists of three independent experiments. Significance was calculated using a two-tailed Student's t-test.

### Resistance to thermal stress

For heat-shock assays, sixty day 1-adult worms on NGM plates seeded with *E. coli* strain HT115 were exposed to 35°C for 4, 5, and 6 hours. Survival was assayed after 14-16 hours of recovery at 20°C. For each time point an independent set of plates was used. Values shown correspond to the average survival of three independent experiments. P values were calculated using a two-tailed Student's t-test.

### RNA extraction, reverse transcription, and qPCR

Total RNA was isolated from synchronized populations of day-1 adult worms using TRIzol (MRC). Reverse transcription was performed using the iScript cDNA Synthesis kit (Bio-Rad) using 500 ng of RNA per sample. SYBR Green real-time qPCR experiments were performed as described in the StepOnePlus manual using a StepOnePlus Real-Time PCR system (Applied Biosystems). PCR products were amplified using the primers listed below (Table 1). The standard curve method was used to determine the relationship between mRNA abundance and PCR cycle number. Levels of *cdc-42* and *pmp-3* mRNA were used for normalization of data shown in Figure 4C; *cdc-42*, *act-1*, and *ama-3* mRNA were used for normalization of data shown in Figure 6A and S5. Each experiment was repeated at least two times using three biological and two technical replicates. ΔCt values were analyzed by unpaired t-test.

**Table 1:** Sequences of primers used for qPCR analysis

Gene	Forward primer	Reverse primer	pAmplicon length (nt)
*cdc-42*	CTGCTGGACAGGAAGATTACG	CTCGGACATTCTCGAATGAAG	111
*pmp-3*	GTTCCCGTGTTCATCACTCAT	ACACCGTCGAGAAGCTGTAGA	115
*sod-3*	TCGCACTGCTTCAAAGCTTGTTCAA	CCAAATCTGCATAGTCAGATGGGAGAT	98
*nhr-57*	TTATCGAGTTTCTCGCATTGG	AAGTCTGCAATCACGCTCTGT	115
*hsp-16.49*	GAGAAATGCTGATCACAACTC	GAAACATCGAGTTGAACAGAG	92
*hsp-16.1*	GGCTCAGATGGAACGTCAA	TGGCAAACTTTTGATCATTGTTA	89
*F22B5.4*	CGCCATTCAGAAGGGAGATA	ATGCACTGCAGAAGAGAACG	119
*ama-1*	CCTACGATGTATCGAGGCAAA	CCTCCCTCCGGTGTAATAATG	139
*act-1*	GCTGGACGTGATCTTACTGATTACC	GTAGCAGAGCTTCTCCTTGATGTC	113

### Western blotting

Approximately 400 L4 worms were collected in M9 buffer and snap frozen in dry ice. Pellets were resuspended in 50 mM Tris, pH 7.5, containing protease inhibitors (Roche). Ceramic beads were added, and worms were lysed using a Precellys 24 homogenizer (Bertin Technologies). Extracts were resolved by SDS-PAGE and transferred to nitrocellulose membrane. Western blot analysis was performed using anti-c-myc (Roche) and anti-α-tubulin (Sigma) primary antibodies. The band intensity was quantified using ImageJ (https://imagej.nih.gov/ij/).

### DAF-16 localization assays

On day 1 of adulthood, animals carrying a *daf-16*::GFP transgene were analyzed for DAF-16 nuclear localization in intestinal cells using a Nikon Eclipse 80i fluorescent microscope at 400x magnification. For the heat-stress challenge, worms were shifted to 37°C for 1 h. Animals were scored as having nuclear DAF-16 if the majority of intestinal cells displayed a distinct concentration of GFP in the nucleus. Approximately 15–30 worms were analyzed for each condition. Each experiment was repeated twice, and a representative experiment is shown in Figure S5

### Analysis of fatty acid composition by GLC

Synchronized L4 stage worms were collected from five to ten 9-cm plates and washed three times in 0.9% NaCl. Worms were left for 20 min to empty their intestines, washed once in sterile water, resuspended in freshly prepared 2.5% (v/v) H_2_SO_4_ in water-free methanol (1 ml) supplemented with 10 μg/ml butylated hydroxytoluene, and incubated for 5 h at 80°C. Subsequently, the fatty acid methyl esters (FAMEs) were extracted by the addition of hexane (0.5 ml) and H_2_O (1.5 ml). The organic phase was transferred to fresh sample vials and dried under a stream of N_2_. Each sample was dissolved in hexane (40–50 μl), and FAMEs were analyzed by GLC on a Chrompack CP 9002 instrument equipped with a DB-WAX column (Agilent Technologies). FAMEs were identified by comparison with standards (Larodan Fine Chemicals).

### Autophagy assay

Autophagy was monitored using an LGG-1::GFP translational reporter [64]. GFP-positive punctae in seam cells were counted in L4 transgenic worms using a Leica DMI 6000 B microscope at 1000× magnification. All animals were kept at 20°C and analyzed by the same experimenter. The number of punctae per seam cell was averaged for each worm and this average was used for calculating the population mean number of LGG-1::GFP-containing punctae per seam cell. Statistical analysis was performed by one-way ANOVA followed by the Dunnett's multiple comparison test.

### ER stress survival assay

L4 worms were placed on plates containing 3 μg/ml tunicamycin (Sigma) and seeded with OP50 bacteria. Eggs laid over 6 h were counted and the number of progeny reaching adulthood was scored after 72 h at 20°C.

### Oil Red O staining

Worms were fixed in 2% paraformaldehyde for 1 h at 20°C, washed twice with PBS, and incubated in 60% isopropanol for 15 min. Worms were stained with a 60% Oil Red O solution overnight, and then observed using an Axioplan microscope (Zeiss) at 100 x magnification.

## SUPPLEMENTARY MATERIAL FIGURES AND TABLES


