# Supplementary material for: HIF-1–dependent regulation of lifespan in Caenorhabditis elegans by the acyl-CoA–binding protein MAA-1
Source: Aging (Albany NY). 2017 Jul 27;9(7):1745–60. doi: 10.18632/aging.101267 (PMC5559173; doi:10.18632/aging.101267)
Supplement: Supplementary file 1 [file aging-09-1745-s001.pdf]

SUPPLEMENTARY MATERIAL

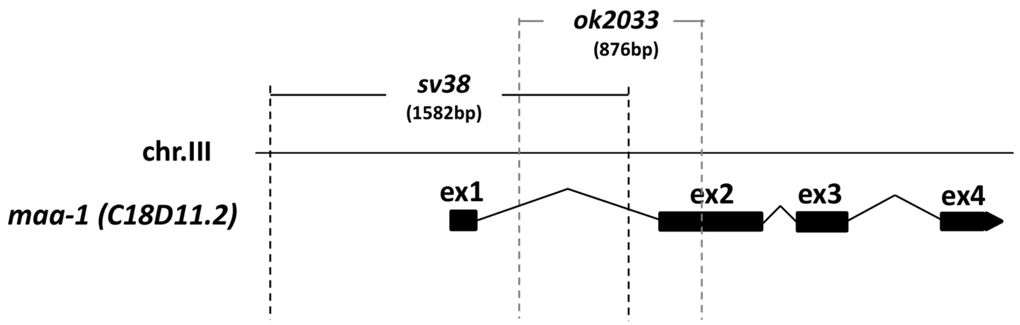

Figure S1. Gene structure of wildtype *maa-1* (C18D11.2), *maa-1(sv38)* and *maa-1(ok2033)* deletion alleles.

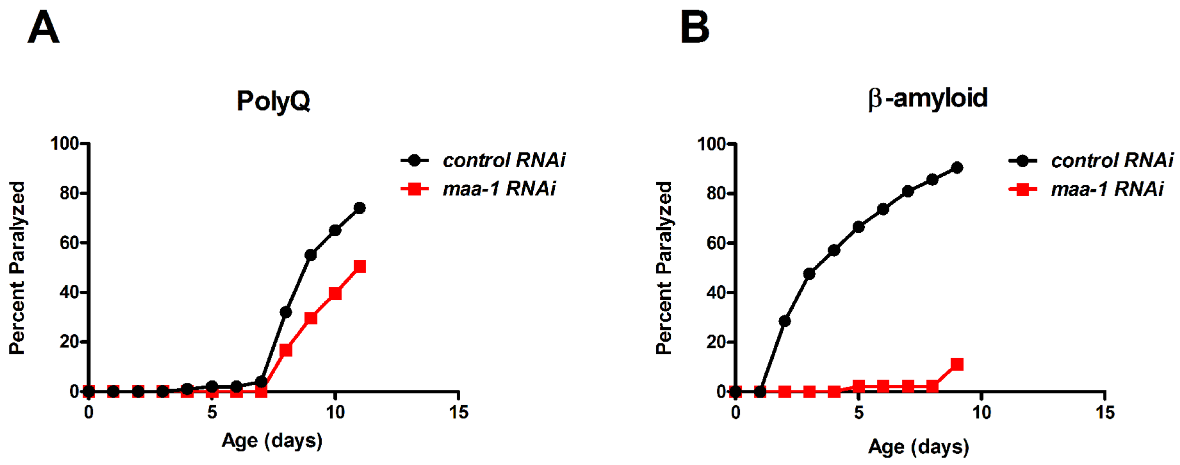

Figure S2. Loss of *maa-1* increases resistance to stress. Repetition of the experiments shown in Figure 2C, D. (**A**-**B**) *maa-1* RNAi increases resistance to paralysis induced by aggregation of a 35-residue polyglutamine repeat protein (**A**) or human β-amyloid (**B**) (P<0.0001 for both **A** and **B**). P values were calculated using the log-rank (Mantel-Cox) method.

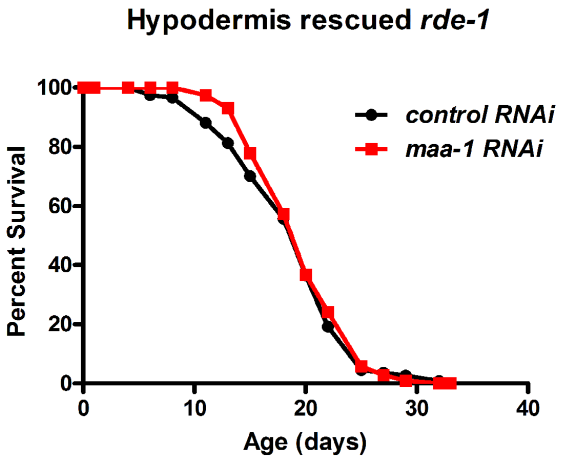

Figure S3. Hypodermal downregulation of *maa-1* does not extend lifespan. Lifespan of *rde-1(ne219)* mutants in which *rde-1* expression is restored in the hypodermis using the *wrt-2* promoter; animals were subjected to control or *maa-1* RNAi (P=0.4960). P values were calculated using the log-rank (Mantel-Cox) method. Replicate experiments and statistical analysis are shown in Table S1 and S2.

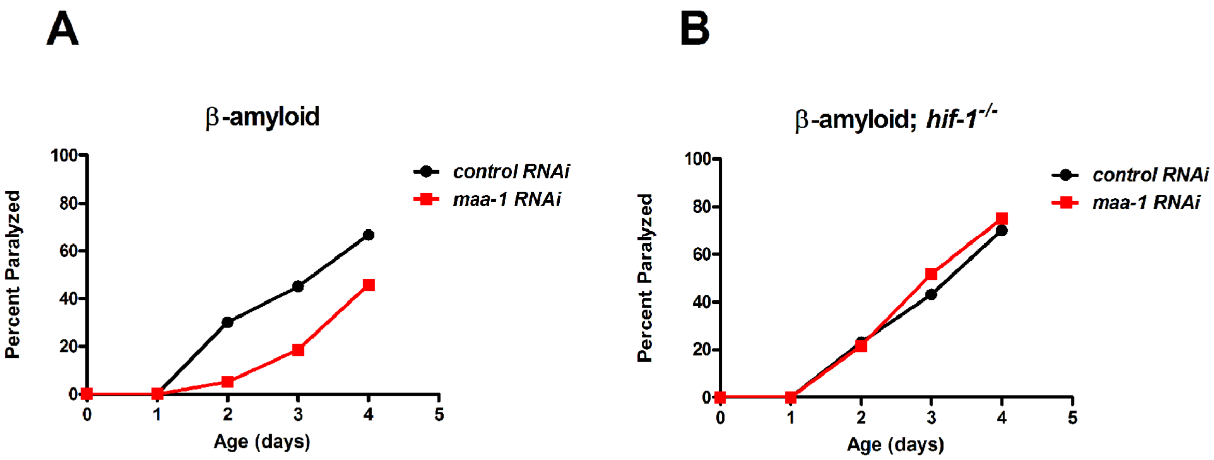

Figure S4. HIF-1 mediates the effect of loss of *maa-1* on proteotoxic stress. (**A**) *maa-1* RNAi increases resistance to paralysis induced by aggregation of human β-amyloid (P<0.0001). (**B**) The effect is absent in transgenic animals lacking *hif-1*. P values were calculated using the log-rank (Mantel-Cox) method.

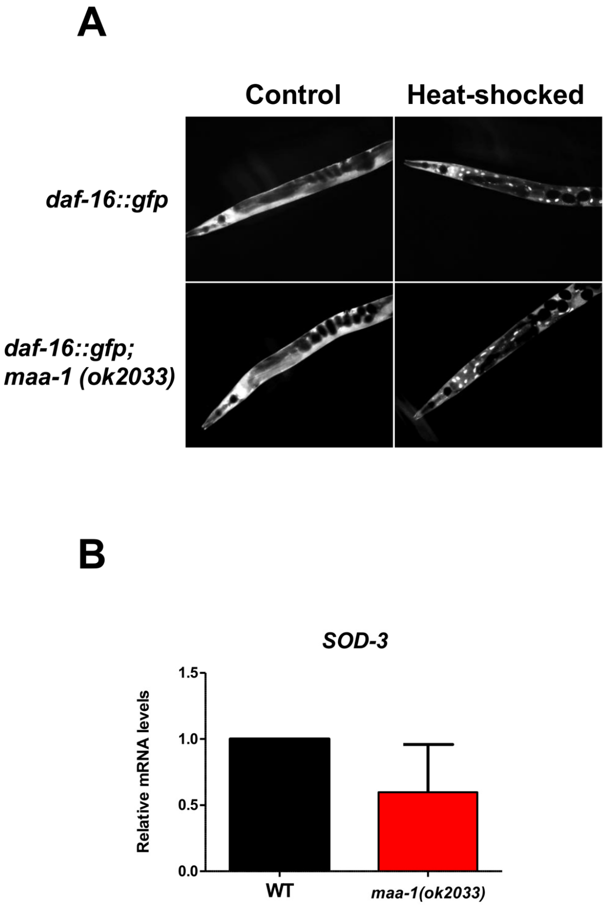

Figure S5. DAF-16 nuclear localization and transcriptional activity are not affected by loss of *maa-1*. (**A**) Localization of DAF-16 in wildtype and *maa-1(ok2033)* mutants expressing a *daf-16::GFP* transgene. Animals were incubated at 20°C (left panels) and at 37°C (right panels). (**B**) qPCR of *sod-3* expression in wild-type and *maa-1(ok2033)* mutants.

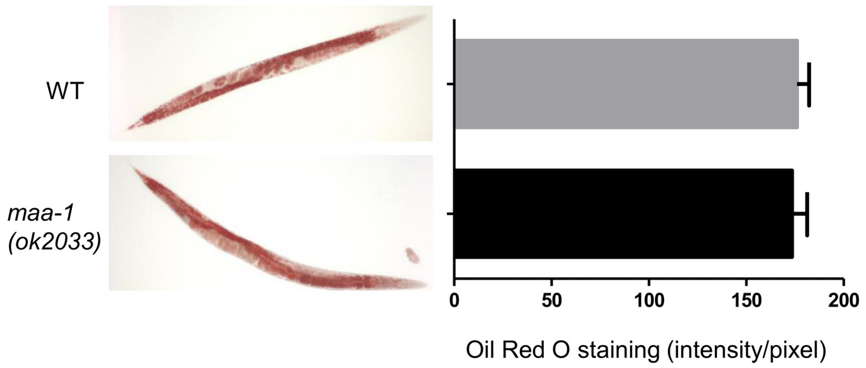

Figure S6. **Total lipid content is not affected by loss of *maa-1*.** Visualization of whole worm total lipid content by Oil Red O staining of wildtype and *maa-1(ok2033)* mutants. Representative pictures are shown in the left panels, and quantification of staining by optical density is shown in the right graph.

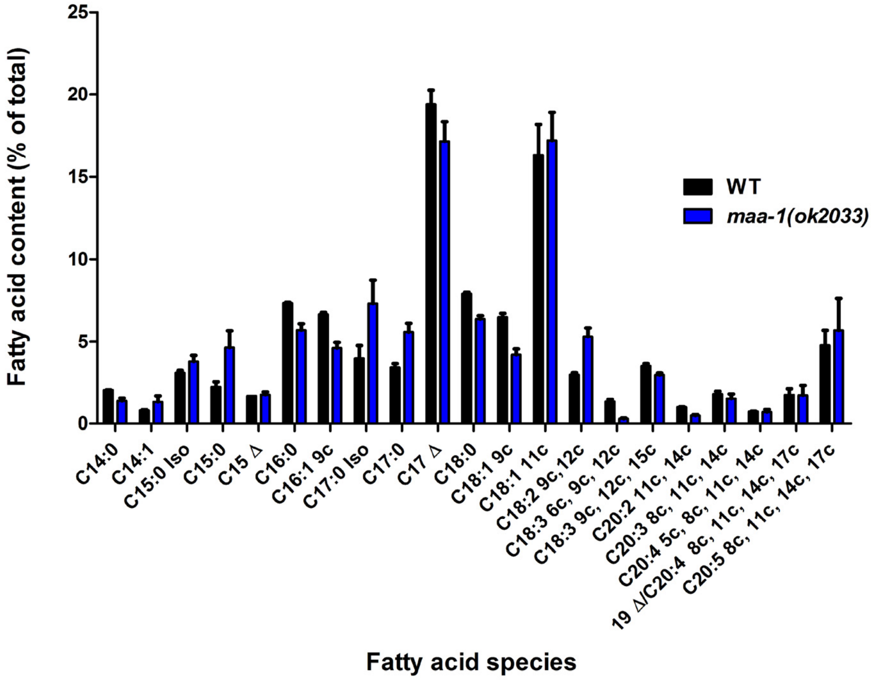

Figure S7. **Loss of MAA-1 does not substantially affect total fatty acid content.** Quantification of fatty acids in wild-type and *maa-1(ok2033)* animals obtained by gas chromatography. Error bars show the standard deviation from three samples obtained from independent preparations.

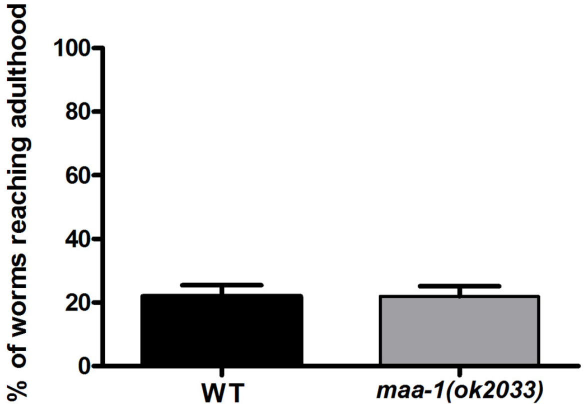

Figure S8. **Loss of *maa-1* does not activate the UPR^{ER}.** Percentage of worms reaching adulthood after 72 h of development from eggs laid on plates containing OP50 bacteria and tunicamycin (3 µg/ml).

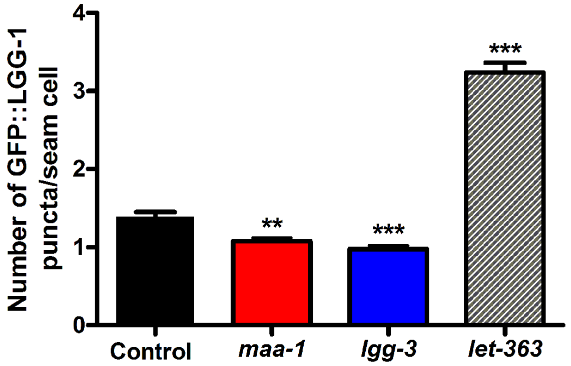

Figure S9. **Autophagy is reduced in response to *maa-1* downregulation.** Quantification of LGG-1::GFP punctae per seam cell of wildtype *C. elegans* subjected to control, *maa-1, lgg-3* (essential for autophagy), or *let-363 (CeTOR)* RNAi (one-way ANOVA: *P<0.05, **P<0.001 vs control RNAi).

Table S1. Summary of adult lifespan data presented in this work.

Strain /Treatment	Maximum Lifespan	Mean Lifespan ± SE (days)	Number of worms (N)	Change (mean lifespan)	P-Value *vs* control	Figure in text
**WT/*control RNAi***	31	20.36 ± 0.51	103			1A
**WT/*maa-1 RNAi***	33	24.29 ± 0.51	89	19%	< 0.0001	1A
**WT/*control RNAi***	30	19.67 ± 0.49	123			Not shown
**WT/*maa-1 RNAi***	32	21.99 ± 0.44	120	11%	<0.01	Not shown
**WT/*control RNAi***	28	16.84 ± 0.35	109			Not shown
**WT/*maa-1 RNAi***	30	21.39 ± 0.40	120	27%	< 0.0001	Not shown
**WT/*control RNAi***	31	19.88 ± 0.46	122			1B
**WT/*acbp-1 RNAi***	32	21.75 ± 0.50	118	9%	0.0052	1B
**WT/*acbp-3 RNAi***	31	21.71 ± 0.42	113	9%	0.0565	1B
**WT**	30	19.70 ± 0.44	115			1C
maa-1(ok2033)	34	25.12 ± 0.55	104	27%	< 0.0001	1C
**WT**	29	20.78 ± 0.47	105			Not shown
maa-1(ok2033)	33	24.60 ± 0.48	96	18%	< 0.0001	Not shown
**WT**	28	20.88 ± 0.28	183			Not shown
maa-1(ok2033)	33	24.68 ± 0.32	189	18%	< 0.0001	Not shown

**WT**	29	19.49 ± 0.37	120			1D
maa-1(sv38)	35	24.59 ± 0.55	98	26%	< 0.0001	1D
**WT**	28	18.89 ± 0.58	92			Not shown
maa-1(sv38)	32	22.70 ± 0.43	101	20%	<0.001	Not shown
**MR0931/*control RNAi***	27	18.70 ± 0.40	108			3A
**MR0931/*maa-1 RNAi***	32	21.64 ± 0.50	99	15%	< 0.0001	3A
**NR222/*control RNAi***	27	19.23 ± 0.40	100			3B
**NR222/*maa-1 RNAi***	30	20.59 ± 0.43	102	7%	<0.05	3B
**WM27/*control RNAi***	33	20.28 ± 0.46	109			3C
**WM27/*maa-1 RNAi***	30	20.35 ± 0.42	110	0.3%	0.8513	3C
**MR0931/*control RNAi***	30	20.34 ± 0.50	115			Not shown
**MR0931/*maa-1 RNAi***	32	22.39 ± 0.52	112	10%	<0.01	Not shown
**NR222/*control RNAi***	30	22.07 ± 0.43	119			Not shown
**NR222/*maa-1 RNAi***	26	21.82 ± 0.31	113	-1%	0.0702	Not shown
**MR0931/*control RNAi***	22	15.60± 0.34	112			Not shown
**MR0931/*maa-1 RNAi***	25	17.77± 0.30	89	14%	<0.005	Not shown
**JM43/*control RNAi***	33	19.02±0.5	118			S3
**JM43/*maa-1 RNAi***	32	19.96±0.4	118	5%	0.4960	S3
**JM43/*control RNAi***	31	21.66±0.39	144		-	Not shown
**JM43/*maa-1 RNAi***	29	22.06±0.33	133	2%	0.6842	Not shown
**JM43/*control RNAi***	31	22.21±0.4	117		-	Not shown
**JM43/*maa-1 RNAi***	35	23.92±0.54	103	8%	<0.001	Not shown
**WT/*control RNAi***	33	20.20 ± 0.49	119			4A
**WT/*hif-1 RNAi***	33	20.79 ± 0.34	117	3%	0.4459	4A
maa-1(ok2033)/control RNAi	38	25.81 ± 0.53	107	27%	< 0.0001	4A
maa-1(ok2033)/hif-1 RNAi	38	23.76 ± 0.63	112	17% vs WT<br>-8% vs *maa-1*	< 0.0001<br>0.10	4A
**WT**	30	19.56 ± 0.43	125			4B
maa-1(ok2033)	37	24.13 ± 0.47	128	23%	<0.0001	4B

hif-1(ia04)	32	21.48 ± 0.47	129	9%	0.0010	4B
maa-1(ok2033);hif-1(ia04)	30	20.36 ± 0.42	122	4% vs WT<br>-16% vs *maa-1*	0.2661<br><0.0001	4B
**WT**	30	19.34± 0.40	117			Not shown
maa-1(ok2033)	34	23.42 ± 0.56	103	21%	<0.0001	Not shown
hif-1(ia04)	35	22.41 ± 0.50	95	16%	<0.0001	Not shown
maa-1(ok2033);hif-1(ia04)	30	19.22 ± 0.45	103	-0.6% vs WT<br>-18% vs *maa-1*	0.7483<br><0.0001	Not shown
**WT**	30	21.17 ± 0.43	103			Not shown
maa-1(ok2033)	32	23.21 ± 0.41	102	10%	0.0023	Not shown
hif-1(ia04)	33	23.37 ± 0.46	95	10%	0.0007	Not shown
maa-1(ok2033);hif-1(ia04)	28	20.49 ± 0.38	102	-3% vs WT<br>-12% vs *maa-1*	0.0690<br><0.0001	Not shown
**WT**	31	18.86 ± 0.41	103			4E
maa-1(ok2033)	34	24 ± 0.53	101	27%	< 0.0001	4E
vhl-1(ok161)	34	25.14 ± 0.63	77	35%	< 0.0001	4E
maa-1(ok2033);vhl-1(ok161)	34	23.96 ± 0.48	107	27% vs WT<br>-0.2% vs *maa-1*<br>-5% vs *vhl-1*	< 0.0001<br>0.8449<br>0.0692	4E
**WT**	30	19.56 ± 0.43	125			Not shown
maa-1(ok2033)	37	24.13 ± 0.47	128	23%	< 0.0001	Not shown
vhl-1(ok161)	39	26.52 ± 0.50	141	35%	< 0.0001	Not shown
maa-1(ok2033); vhl-1(ok161)	35	24.29 ± 0.48	125	24% vs WT<br>0.6% vs *maa-1*<br>-8% vs *vhl-1*	< 0.0001<br>0.8602<br><0.001	Not shown
**WT/*control RNAi***	30	19.56 ± 0.43	125			4F
**WT/*maa-1 RNAi***	31	22.48 ± 0.44	102	15%	<0.001	4F
hif-1 OE	33	26.96 ± 0.45	98	38%	< 0.0001	4F
hif-1 OE/maa-1 RNAi	33	25.91 ± 0.47	102	32% vs WT<br>-4% *vs hif-1 OE*	< 0.0001<br>0.067	4F
**WT/*control RNAi***	25	16.57 ± 0.43	118			Not shown
**WT/*maa-1 RNAi***	30	18.88±0.42	124	13%	<0.001	Not shown
hif-1 OE	32	23.54 ± 0.39	109	42%	< 0.0001	Not shown
hif-1 OE/maa-1 RNAi	32	24.59 ± 0.37	108	48%<br>4% *vs hif-1 OE*	< 0.0001<br>0.0981	Not shown

**WT**	28	20.88 ± 0.28	183			5
maa-1(ok2033)	33	24.68 ± 0.32	189	18%	< 0.0001	5
daf-16(mu86)	22	16.07 ± 0.29	100	-23%	< 0.0001	5
maa-1(ok2033);daf-16(mu86)	22	15.06 ± 0.23	98	-28% vs WT<br>-39% vs *maa-1*	< 0.0001<br><0.0001	5
daf-16(mu86)	24	17.17 ± 0.38	103			Not shown
maa-1(ok2033);daf-16(mu86)	26	18.26 ± 0.39	97	6% vs *daf-16*	0.0396	Not shown
**WT**	33	18.17±0.42	115			Not shown
maa-1(ok2033)	35	22.14±0.49	124	22%	< 0.0001	Not shown
daf-16(mu86)	25	16.71±0.21	121	-8%	< 0.005	Not shown
maa-1(ok2033);daf-16(mu86)	25	17.04±0.19	114	-6% vs WT<br>-23% *vs maa-1*	0.0137<br>< 0.0001	Not shown
**WT/*control RNAi***	28	19.60± 0.52	140			6B
**WT/*hsp-16.1 RNAi***	28	18.64 ± 0.48	161	-5%	0.1024	6B
**WT/*hsp-16.49 RNAi***	28	18.78± 0.52	160	-4%	0.2988	6B
maa-1(ok2033)/control RNAi	36	25.80 ± 0.52	133			6C
maa-1(ok2033)/hsp-16.1 RNAi	32	19.76 ± 0.63	146	-23%<br>*vs maa-1*	< 0.0001	6C
maa-1(ok2033)/hsp-16.49 RNAi	32	18.81 ± 0.59	158	-27%<br>*vs maa-1*	< 0.0001	6C
**WT/*control RNAi***	32	19.54 ± 0.63	153			Not shown
**WT/*hsp-16.1 RNAi***	27	17.93 ± 0.56	152	-8%	<0.001	Not shown
**WT/*hsp-16.49 RNAi***	29	18.18 ± 0.60	160	-7%	0.0078	Not shown
maa-1(ok2033)/control RNAi	33	22.93 ± 0.52	160			Not shown
maa-1(ok2033)/hsp-16.1 RNAi	31	19.66 ± 0.49	160	-14%<br>*vs maa-1*	< 0.0001	Not shown
maa-1(ok2033)/hsp-16.49 RNAi	31	19.14 ± 0.53	140	-17%<br>*vs maa-1*	< 0.0001	Not shown

**WT/*control RNAi***	28	18.89±0.58	97			Not shown
**WT/*hsp-16.1 RNAi***	30	17.44±0.48	95	-8%	0.0838	Not shown
**WT/*hsp-16.49 RNAi***	26	18.44±0.55	86	-2%	0.5796	Not shown
maa-1(ok2033)/control RNAi	32	23.66±0.60	79			Not shown
maa-1(ok2033)/hsp-16.1 RNAi	30	19.08±0.53	98	-19% *vs maa-1*	< 0.0001	Not shown
maa-1(ok2033)/hsp-16.49 RNAi	30	20.12±0.74	60	-15% *vs maa-1*	< 0.05	Not shown

Table S2. Results of two-tailed t-test performed on replicate experiments shown in Table S1.

Comparison	N (number of experiments)	Average change (mean lifespan)	P-value (mean life span)	P-value (maximum lifespan)
**WT/*control RNAi* vs WT/*maa-1 RNAi***	5	17%	<0.005	<0.05
**WT *vs maa-1(ok2033)***	10	20.7%	<0.0001	<0.0001
**MR0391/*control RNAi vs* MR0931/*maa-1 RNAi***	3	13%	<0.05	0.0634
**JM43/*control RNAi vs* JM43/*maa-1 RNAi***	3	5%	0.1106	0.8740
**WT *vs maa-1(ok2033);hif-1(ia04)***	3	0.4%	1	1
maa-1(ok2033) vs maa-1(ok2033);hif-1(ia04)	3	-15.3%	<0.05	<0.05
**WT *vs hif-1(ia04)***	3	11.7%	<0.05	0.0634
maa-1(ok2033);hif-1(ia04) vs hif-1(ia04)	3	12%	0.0672	0.0572
**WT *vs maa-1(ok2033);daf-16(mu86)***	2	-17.1%	0.3701	0.3500
maa-1(ok2033) vs maa-1(ok2033);daf-16(mu86)	2	-31%	0.1891	<0.05

**WT** ***vs daf-16(mu86)***	2	-15.5%	0.3072	0.0903
maa-1(ok2033);daf-16(mu86) vs daf-16(mu86)	3	0.7%	0.8477	0.4226
hif-1 OE vs hif-1 OE/maa-1 RNAi	2	0	1	1
vhl-1(ok161) vs maa-1(ok2033); vhl-1(ok161)	2	-6.5%	0.2048	0.5
**WT/*control RNAi vs*** **WT/*hsp-16.1 RNAi***	3	-7%	<0.05	0.7418
**WT/*control RNAi vs*** **WT/*hsp-16.49 RNAi***	3	-4.3%	0.0801	0.4226
maa-1(ok2033)/control RNAi vs maa-1(ok2033)/ hsp-16.1 RNAi	3	-18.7%	<0.05	0.0572
maa-1(ok2033)/control RNAi vs maa-1(ok2033)/ hsp-16.49 RNAi	3	-19.7%	0.0501	0.0572
